# Chemical Synthesis of Monolignols: Traditional Methods, Recent Advances, and Future Challenges in Sustainable Processes

**DOI:** 10.3390/antiox13111387

**Published:** 2024-11-14

**Authors:** Davide Benedetto Tiz, Giorgio Tofani, Filipa A. Vicente, Blaž Likozar

**Affiliations:** 1 Department of Catalysis and Chemical Reaction Engineering, National Institute of Chemistry, Hajdrihova 19, 1000 Ljubljana, Slovenia; giorgio.tofani@ki.si (G.T.); blaz.likozar@ki.si (B.L.); 2 Faculty of Chemistry and Chemical Technology, University of Ljubljana, Večna pot 113, 1000 Ljubljana, Slovenia

**Keywords:** monolignols, chemical synthesis, *p*-coumaryl alcohol, coniferyl alcohol, sinapyl alcohol, lignin

## Abstract

Monolignols represent pivotal alcohol-based constituents in lignin synthesis, playing indispensable roles in plant growth and development with profound implications for industries reliant on wood and paper. Monolignols and their derivates have multiple applications in several industries. Monolignols exhibit antioxidant activity due to their ability to donate hydrogen atoms or electrons to neutralize free radicals, thus preventing oxidative stress and damage to cells. Characterized by their alcohol functionalities, monolignols present three main forms: *p*-coumaryl alcohol, coniferyl alcohol, and sinapyl alcohol. In nature, particularly in plants, monolignols with geometry (*E*) predominate over their *Z* counterparts. The methods for obtaining the three canonical monolignols, two less-common monolignols, and a monolignol analogue are addressed to present an overview of these phenol-based compounds, particularly from a synthetic standpoint. A SWOT (Strengths, Weaknesses, Opportunities, and Threats) analysis is used to explain the advantages and disadvantages of synthesizing monolignols, key alcohol-containing raw materials with enormous significance in both plant biology and industrial applications, using bench chemical methods. The uniqueness of this work is that it provides an overview of the synthetic pathways of monolignols to assist researchers in pharmaceutical and biological fields in selecting an appropriate procedure for the preparation of their lignin models. Moreover, we aim to inspire scientists, particularly chemists, to develop more sustainable synthetic protocols for monolignols.

## 1. Introduction

**Figure 1:**
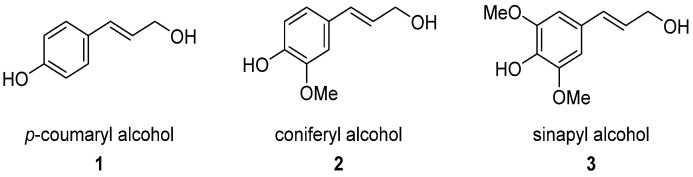
Chemical structure of the three monolignols found in lignin: *p*-coumaryl alcohol, coniferyl alcohol, and sinapyl alcohol.

Monolignols (*p*-coumaryl alcohol **1**, coniferyl alcohol **2**, and sinapyl alcohol **3**, Figure 1) and their analogues have attracted significant interest from researchers due to their potential applications in the production of renewable materials and biofuels, such as bioethanol and biodiesel [1,2]. They can also be used in the production of various chemicals, including fragrances, flavors, and pharmaceuticals [3].

In pharmaceutics, some monolignols and their resulting derivates have shown antioxidant and anti-inflammatory properties and may have potential applications in the prevention and treatment of certain diseases. A derivative of *p*-coumaryl alcohol, *p*-coumaryl alcohol-γ-O-methyl ether (CAME), was isolated from *Alpinia galanga* and revealed to contain a phenylpropanoid structure similar to *p*-coumaryl diacetate (CDA). CDA is known to have antioxidant and anti-inflammatory activity [4]. Coniferyl alcohol was studied as an inhibitor of cell growth of cholangiocarcinoma [5]. The cytotoxicity of sinapyl alcohol derivatives was studied using tumor cells for anti-tumoral applications [6,7]. Moreover, monolignols and monolignol derivatives found applications as building blocks of pesticides, allowing them to obtain more natural and sustainable products due to the biocompatibility of the monolignols with respect to fossil-based pesticides [8,9]. Overall, the potential applications of monolignols are diverse and could have a significant impact on industries ranging from energy, materials, and agriculture to health. However, further research is needed to access the full potential of these compounds.

Monolignols are the building blocks of lignin, the richest source of aromatics on earth. These are derived from the general phenylpropanoid biosynthetic pathway, starting from the amino acids phenylalanine [10,11] and tyrosine [12,13]. Beginning with the deamination of phenylalanine or tyrosine, monolignol biosynthesis entails, in short, the sequential hydroxylation reactions of the aromatic ring, phenolic O-methylation, and the reduction of the carboxylic acid group on the side chain via a variety of enzymes to an aldehyde, and ultimately to an alcohol [14]. The percentage of each monolignol is ontogenetic-tissue-species dependent [15,16]. In softwoods, coniferyl alcohol is 90% predominant [17]. The chemical composition of hardwood lignin is given by mainly coniferyl alcohol and sinapyl alcohol units [18]. Other phenolic compounds originating from pathways other than the canonical monolignol biosynthetic one, such as the flavonoid hydroxystilbene or the hydroxycinnamamide biosynthetic pathways, have also been demonstrated to behave as lignin monomers [19]. Looking at the chemical properties of monolignols, like other compounds with a C=C double bond, they can have two geometric isomers: the *E* (trans) and the *Z* (cis) form. In the *E*-configuration, the substituent groups are arranged opposite to each other around the double bond, and in the *Z*-configuration, they are on the same side. In monolignols, the *E*-configuration is usually more stable at the double bond (between the α and β carbons in the side chain) and is the typical form present during lignin biosynthesis. This configuration is generally preferred due to the lower steric hindrance and lower energy associated with the arrangement of substituents around the double bond, but there is evidence of isomerization in the literature. For instance, photoirradiation can convert (*E*) monolignols to the corresponding (*Z*) derivatives [20]. Isomerism between the two geometries has also been proposed in vivo. A study on glucosyltransferase activity exhibits a very unusual substrate specificity for (*Z*) and not (*E*) monolignols [21]. (Z)-*p*-coumaryl alcohol has been identified in *Angelica keiskei* (Umbelliferae), a plant found in Asia, mainly in Japan and Korea [22]. Gala apples contain both (*E*) and (*Z*) isomers of *p*-coumaryl alcohol with primarily long-chain, saturated fatty acids esterified at the primary alcoholic group [23].

Three pathways can be considered to obtain the three main monolignols: depolymerization from lignin, biosynthesis, and chemical synthesis. With the European Green Deal targeting climate neutrality by 2050 [24], more focus has been directed towards the use of renewable resources, including the “upstream” and “downstream” valorization of lignin. However, the lignin oxidative depolymerization is limited to producing lignin monomers, namely aromatic aldehydes like vanillin and syringaldehyde, as well as related acids like vanillic acid and syringic acid [25]. The reductive depolymerization of lignin can generate monolignol derivatives [26]. This has only been performed for analytical studies at a laboratory scale; for example, Khan et al. [26] operated using a 50 mL Parr reactor. The problem of recovering monolignols is caused by the highly branched structure of lignin, but the same issue is also observed in a unique and particular type of lignin called catechyl lignin, or poly-(caffeyl alcohol), which is a linear homopolymer found in seeds [27,28,29,30]. In fact, caffeyl alcohol is not extracted and recovered, despite the extensive research that has been performed [31,32,33,34,35]. The main limit is the modification of the monolignols during lignin biosynthesis to form the polymeric structure, which leads to the formation of covalent bonds and, during depolymerization, to the formation of complex mixtures of phenolic compounds that are subsequently difficult to separate and purify. As lignin is difficult to break down due to its complex structure and strong C–C bonds, its degradation has recently been carried out under relatively mild conditions (e.g., sodium persulphate as an oxidizing agent), which are more energy efficient and potentially more environmentally friendly than conventional methods [36]. However, it should be highlighted that monolignols are lignin monomers, but not all lignin monomers are monolignols. The term lignin monomer refers to any unit that takes part of in the synthesis of lignin (monolignols) and also the units generated during lignin depolymerization such as vanillin. These modifications (lignin biosynthesis and depolymerization) create a variety of substructures within the lignin, resulting in different binding patterns and functional groups beyond the original monolignol forms. This distinction is crucial for understanding the complexity and variability of lignin derivatives, starting from different plant species and extraction methods. In conclusion, monolignols from lignin cannot currently be recovered; only some monolignol derivatives, which are better known as lignin monomers. Another homopolymer lignin composed of 5-hydroxyconiferyl alcohol units is also present in seed coats [37] but, to the best of our knowledge, no valorization pathways have been studied until now.

Regarding monolignol biosynthesis, genetically modified organisms represent an opportunity. Yet, these come with a higher production cost and usually low yields. For instance, *Escherichia coli* cells equipped with an artificial chimeric phenylpropanoid pathway could provide an alternative source of *p*-coumaryl alcohol that does not require the inefficient decomposition of lignin [38]. Jansen et al. [39] succeeded in preparing *p*-coumaryl alcohol by transferring several genes from plants and microbes to *Escherichia coli* cells. The established chimeric pathway efficiently converts L-tyrosine into the lignin precursor molecule. However, cultivation in a minimal growth medium resulted in very low product yields, which did not improve with incubation times. Another biocatalysis approach was used by Liu et al. [40], where *p*-coumaryl alcohol and coniferyl alcohol were produced using immobilized whole cells of engineered *Escherichia coli* as the biocatalyst. The molar yields of *p*-coumaryl alcohol and coniferyl alcohol were 58% and 60%, respectively. More recently, Zhao et al. [41] engineered several whole-cell bioconversion systems with carboxylate reductase-mediated pathways to synthesis *p*-coumaryl, caffeyl, and coniferyl alcohols from L-tyrosine in *Escherichia coli* BL21 (DE3). The authors achieved the production of ~1028 mg L^−1^ of *p*-coumaryl alcohol, ~1015 mg L^−1^ of caffeyl alcohol, and ~411 mg L^−1^ of coniferyl alcohol, corresponding to productivities of 257 mg L^−1^ h^−1^, 203 mg L^−1^ h^−1^, and 82 mg L^−1^ h^−1^, respectively. Another approach consists in the bioconversion of eugenol to produce coniferyl alcohol using a recombinant strain of *Saccharomyces cerevisiae* [42]. However, despite these efforts, two major challenges continue to hinder the study of monolignol biosynthetic pathways and the development of downstream applications for these valuable metabolites. These challenges are the lack of commercial availability for all 24 essential metabolites and the complexity of synthesizing them using chemical, enzymatic, or biosynthetic methods. Nonetheless, some progress has been made, as demonstrated by Kao et al. [43], who recently identified the biosynthetic pathways for all these metabolites in planta, specifically in *Populus trichocarpa* and *Eucalyptus grandis*. In this regard, mathematical models and computational simulations have recently shown significant potential for advancing our understanding of the fundamental metabolism of lignin and related phenolic compounds [44]. However, developing a comprehensive and accurate model of the lignin metabolic network remains challenging. To improve their predictive power, future models should integrate precise data on the temporal and spatial variability of lignin formation, encompassing the transport, storage, signaling, and regulatory processes that drive monolignol biosynthesis in vivo.

Overall, monolignols’ natural biosynthesis, their production via microbial systems (e.g., *Escherichia coli* [45]), and their extraction from biomass are interesting options to obtain these compounds and to study their fundamental role in several fields [45,46] however, this is not always possible or an easy task. Therefore, the development of green synthetic routes for monolignols has been an active area of research in recent years. New synthetic methods have been developed using green chemistry concepts, including the use of renewable raw materials, non-toxic reagents and solvents, and reducing waste and energy usage [47,48]. However, it is important to note that not all synthetic routes for monolignols are green, and some may involve toxic reagents, solvents, or energy-intensive processes that can have negative environmental impacts. Therefore, it is essential to carefully evaluate the environmental impact of each synthetic route and choose the most sustainable option available. Wittig and Horner–WadsworthEmmons (HWE) reactions are typical reaction pathways used to synthesize monolignols. In this reaction, an olefin is created by treating an aldehyde with an ylide or phosphonate ester, respectively. Sinapaldehyde, coniferyl aldehyde, and *p*-coumaric acid can all be produced using this reaction and reduced to the corresponding alcohols. Palladium-catalyzed reactions (e.g., Fujiwara–Moritani reactions) are another way to make monolignols. In the Suzuki coupling, a new C–C bond is created when an aryl halide reacts with boronic acid in the presence of a palladium catalyst in this reaction. The Fujiwara–Moritani reaction is a type of cross-coupling reaction where an aromatic C–H bond is directly coupled to an olefinic C–H bond, generating a new C–C bond. Coniferyl alcohol and sinapyl alcohol are just two of the monolignols that can be created via this process. In addition to these methods, there are many other organic synthesis strategies that can be used to produce monolignols. These methods often involve protecting and deprotecting various functional groups, as well as forming and breaking carbon–carbon bonds using a variety of chemical reactions. The specific synthesis strategy used will depend on the desired monolignol product and the starting materials available.

This manuscript describes the synthesis of the three main monolignols and the preparation of two less-common monolignols, namely caffeyl alcohol and 5-hydroxyconiferyl alcohol, as well as the monolignol analogue iso-sinapyl alcohol. This review aims to provide a clear overview of the synthetic protocols that are currently used for monolignols for two reasons. Firstly, it will support researchers in pharmaceutical and biological fields who are currently studying or want to study monolignols by offering a list of synthetic pathways that can be selected and reproduced in their laboratories to prepare small-scale samples for their experiments [26]. Secondly, it will inspire scientists, in particular chemists, to be involved in the transition from fossil-based to green processes by demonstrating the advantages and weaknesses of the synthesis of monolignols.

## 2. Synthetic Approaches to Prepare Monolignols

The synthetic preparation of the three monolignols will be discussed in the next paragraphs. The reaction conditions and information about regioselectivity and yields (when available) will be provided. The yield of monolignols in organic synthesis can vary depending on a number of factors, such as the specific reaction conditions used, the starting materials employed, and the purification methods employed. The chemical structures of the monolignols, precursors, catalysts, and reagents described in this paper are shown in Table 1. Overall, the choice of synthetic approach will depend on the desired quantity, purity, and cost-effectiveness of the monolignol product.

### 2.1. p-Coumaryl Alcohol

As previously mentioned, *p*-coumaryl alcohol is one the main monolignols. It displays an aromatic alcohol in the *para* position to an allyl chain. As a natural polyphenol, it presents antioxidant activity that is commonly attributed to its hydroxyl groups. As primary antioxidants, polyphenols inactivate free radicals according to the hydrogen atom transfer and to the single electron transfer mechanisms [49]. In the case of *p*-coumaryl alcohol, there is a single phenolic hydroxyl group at an aromatic ring in the *para*-position to a conjugated allyl side chain. This *para*-substitution and the conjugated double bond allow the corresponding phenoxyl radical to be highly delocalized. Upon modification, for instance, to *p*-coumaryl alcohol 4-O-glucoside, its antioxidant activity enhances considerably, as well as its anti-inflammatory activity, hence explaining its wide application in the cosmetic and pharmaceutical industries [50].

**Scheme 1:**
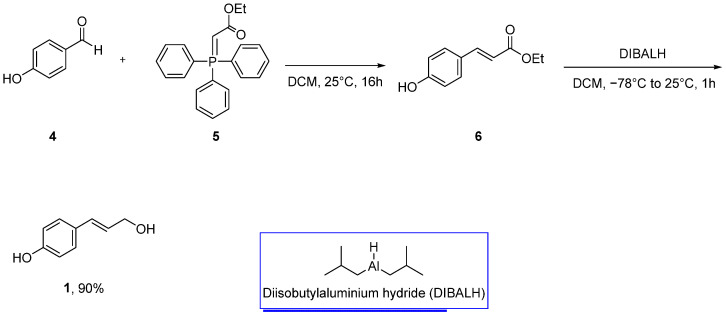
Synthesis of *p*-coumaryl alcohol via Wittig reaction. The structure of DIBALH is reported in the blue box.

A classical way to obtain *p*-coumaryl alcohol is the Wittig reaction between 4-hydroxybenzaldehyde (**4**) and ethyl(triphenylphosphoranylidene)acetate (**5**) to give the corresponding (*E*)-alkene coumarate **6**. The reduction mediated by diisobutylaluminium hydride (DIBALH) in dichloromethane afforded paracoumaryl alcohol **1** with a 90% yield (Scheme 1) [51].

**Scheme 2:**
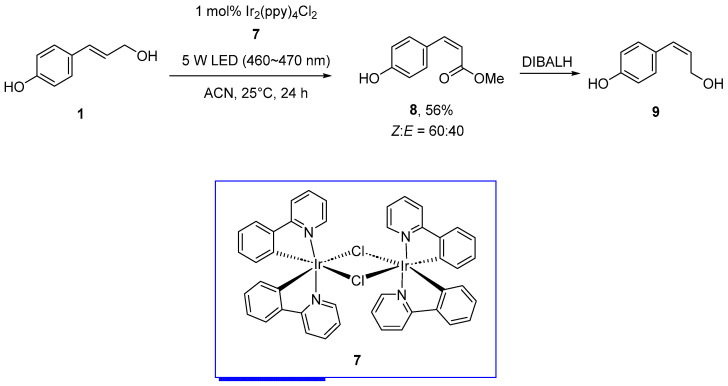
Synthesis of (*Z*)-*p*-coumaryl alcohol **9** via blue LED activation pathway. The structure of Ir_2_(ppy)_4_Cl_2_ (**7**) is reported in the blue box.

The preparation of allylic alcohols from α and β-unsaturated carboxylic esters using LiAlH_4_/BnCl has been reported by Wang et al. [52]. Starting from **6**, they obtained product **1** with an 83% yield. The mixture of LiAlH_4_/alkyl halide methodology was used for generating AlH_3_ as a reducing agent. THF was used as a solvent. The corresponding (*Z*)-coumarate (**8**) was prepared by treating *p*-coumaryl alcohol (**1**) in acetonitrile with blue LED light and an iridium catalyst **7**, Ir_2_(ppy)_4_Cl_2_. Intermediate **8** can eventually be converted to (*Z*)-*p*-coumaryl alcohol **9** by DIBALH. The preparation of **8** is mild, with the conversion requiring simple, green conditions, resulting in a good yield (56%) and with a *Z*/*E* ratio of 60/40 (Scheme 2) [53].

**Scheme 3:**
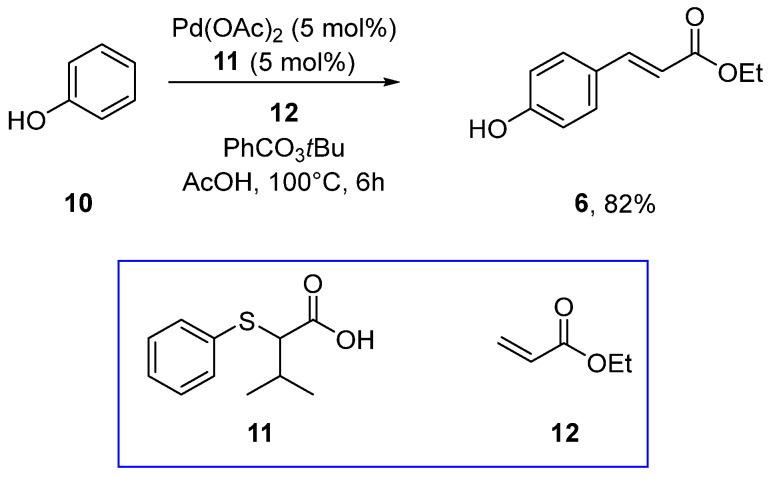
Palladium-catalyzed activation to obtain intermediate **6**, useful for the synthesis of *p*-coumaryl alcohol. 3-methyl-2-(phenylthio)butanoic acid (**11**) and ethyl acrylate (**12**) are displayed in the blue box.

The preparation of intermediate **6** has been reported directly from the bare phenol **10** via palladium-catalyzed activation (Scheme 3) [54]. The reaction conditions employ palladium (II) acetate as a catalyst, 3-methyl-2-(phenylthio)butanoic acid (**11**) as a ligand, ethyl acrylate (**12**), and tert-butyl peroxybenzoate in acetic acid as a solvent, affording a mixture of *ortho* and *para* derivatives (ratio of 1.9:1, respectively).

**Scheme 4:**
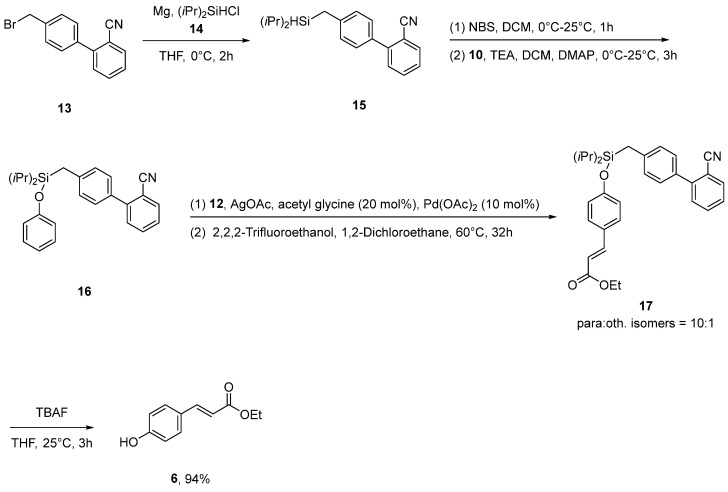
Preparation of **6** via Pd-catalyzed activation (see text for more details).

Another reported way for preparing **6** has been the activation of the phenolic group with a silicon-containing *para*-directing moiety followed by a palladium-catalyzed Fujiwara−Moritani reaction with ethyl acrylate (Scheme 4) [55]. The synthesis starts from the nitrile-containing biphenyl derivative **13**, which is converted into a nucleophilic species via the addition of magnesium turnings and reacted with chlorodiisopropylsilane **14** to afford the silicon-based species **15**. The bromination provided by *N*-bromosuccinimide (NBS) at the silicon center followed by nucleophilic attack of phenol (**10**) in presence of triethylamine as base and 4-dimethylaminopyridine (DMAP) as a catalyst provided **16**. The treatment of **16** with an oxidant (silver acetate, AgOAc), acetyl glycine as a ligand, palladium acetate as a catalyst, and ethyl acrylate 12 in 2,2,2-trifluoroethanol, 1,2-dichloroethane (TFE/DCE) as solvents provided the preferential *pa*ra-substituted derivative **17** (ratio of 10:1 with respect to other obtained isomers). The selective removal (tetra-*n*-butylammonium fluoride, TBAF) in THF of the silicon-based pendant yielded (*E*)-coumarate **6** with an excellent yield (94%).

### 2.2. Coniferyl Alcohol

**Scheme 5:**
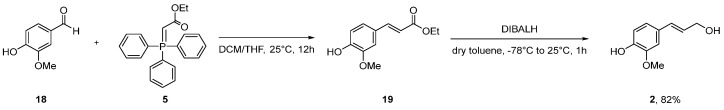
Synthesis of coniferyl alcohol **2** via Wittig reaction.

Coniferyl alcohol (**2**, Figure 1) differs from *p*-coumaryl alcohol due to the presence of a methoxy group in the *ortho* position to the phenolic function, with vanillin being the precursor for compound **2**. Coniferyl alcohol is considered to a potent antioxidant and a precursor of several bioactive products [56]. The antioxidative activity of polyphenols is generally attributed to their hydroxyl groups. Coniferyl alcohol and coniferyl thiol have an aromatic ring with a single phenolic hydroxyl group in the *para*-position involving a conjugated allyl side chain. The conjugated double bond and its *para*-substitution enable a high degree of delocalization for the associated phenoxyl radical [56]. Similarly to the preparation of *p*-coumaryl alcohol (Scheme 1), the synthesis of coniferyl alcohol involves a Wittig reaction between vanillin (4-hydroxy-3-methoxybenzaldehyde, **18**) and ethyl(triphenylphosphoranylidene)acetate (**5**) to afford the ethyl ester **19**, which was reduced by DIBALH to coniferyl alcohol (**2**) with a good yield (82%, Scheme 5) [57].

**Scheme 6:**
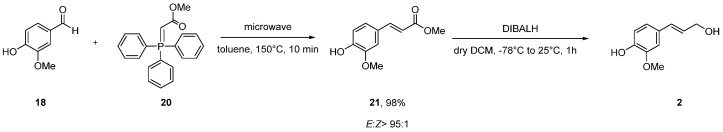
Microwave-assisted synthesis of coniferyl alcohol **2** via Wittig reaction.

A similar procedure has been proposed by Konrádová et al. [58] but using a microwave-assisted protocol. The synthesis was carried out in toluene at 150 °C, employing vanillin (**18**) and Wittig ylide **20** to afford methyl ester derivative **21** with an excellent yield (98%, regioselectivity *E*/*Z* > 95/1). This was then reduced by DIBALH to give coniferyl alcohol **2** (Scheme 6).

**Scheme 7:**
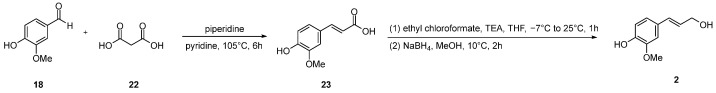
Synthesis of coniferyl alcohol **2** via Knoevenagel condensation.

An alternative synthetic pathway (Scheme 7) is given by Knoevenagel condensation between vanillin (**18**) and malonic acid **22** in the presence of piperidine as a base. The obtained carboxylic acid intermediate (ferulic acid, **23**) was then converted into carboxylic anhydride and lastly reduced to give alcohol **2** via sodium borohydride in methanol [59]. The phenolic nucleus of ferulic acid, together with an extended side chain conjugation, account for a resonance-stabilized phenoxy radical, which accounts for its potent antioxidant potential [60]. A plethora of other biological activities, such as anti-inflammatory, antimicrobial, antiallergic, hepatoprotective, anticarcinogenic, and antithrombotic, are exhibited by ferulic acid [61]. Ferulic acid exhibits anti-inflammatory effects by inducing autophagy; the natural, conserved degradation of a cell that removes unnecessary or dysfunctional components through a lysosome-dependent regulated process [62]. The antimicrobial effect could be ascribed to it causing cell membrane dysfunction and changes in cellular morphology [44]. The antiallergic properties are due to attenuated eosinophilic pulmonary infiltration in a dose-dependent manner, among others [63]. The hepatoprotective effect has been compared to that of the drug silymarin, as evidenced in liver histology [64]. Inhibition of cell proliferation and promotion of apoptosis account for its anticarcinogenic activity in rats [65]. Antithrombotic activity is connected not only to the inhibition of platelet aggregation but also to the protection of the vascular endothelial cells [66].

**Scheme 8:**
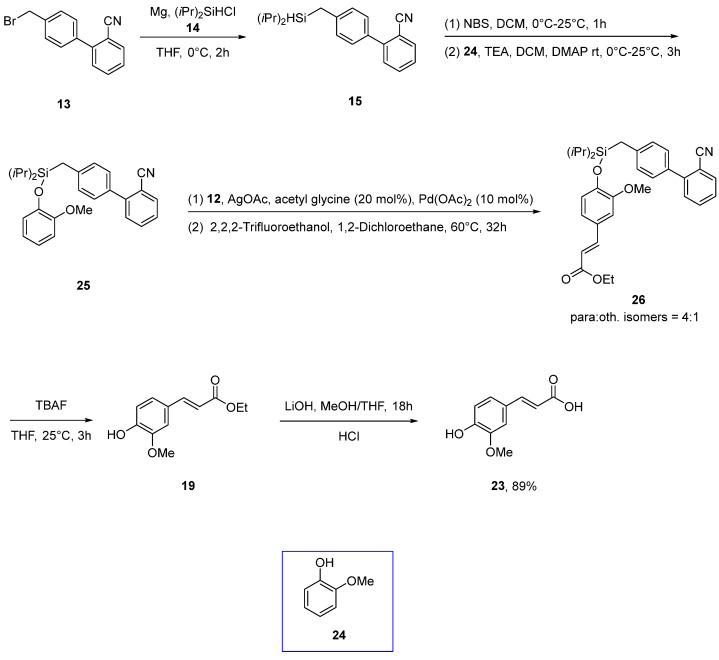
Preparation of ferulic acid **23** via Pd-catalyzed activation preceded by installation of a silicon-containing group on phenolic oxygen (see text for more details). The structure of guaiacol (**24**) is reported in the blue box.

As seen for *p*-coumaryl alcohol, the synthesis of ferulic acid (**23**) using the silicon-containing pendant as an activating moiety of phenolic oxygen [55] has been provided. Instead of the bare initial phenol, guaiacol (**24**) was employed. The formation of guaiacol-derivative **25** was followed by Pd-catalyzed olefination with **12** to afford para-derivative **26** (selectivity ratio of 4:1 with respect to other obtained isomers). The removal of the silicon-directing moiety by TBAF in THF provided **19**. The ethyl ester **19** was followed by alkaline hydrolysis to give ferulic acid (**23**) with a very good yield (89%, Scheme 8).

A recently reported photoredox system afforded ferulic acid (**23**) in a single-step Fujiwara−Moritani reaction [46]. In particular, the reaction involves a palladium/organo-photocatalyst that forges oxidative olefination in a regioselective fashion with diverse types of arenes and heteroarenes.

**Scheme 9:**
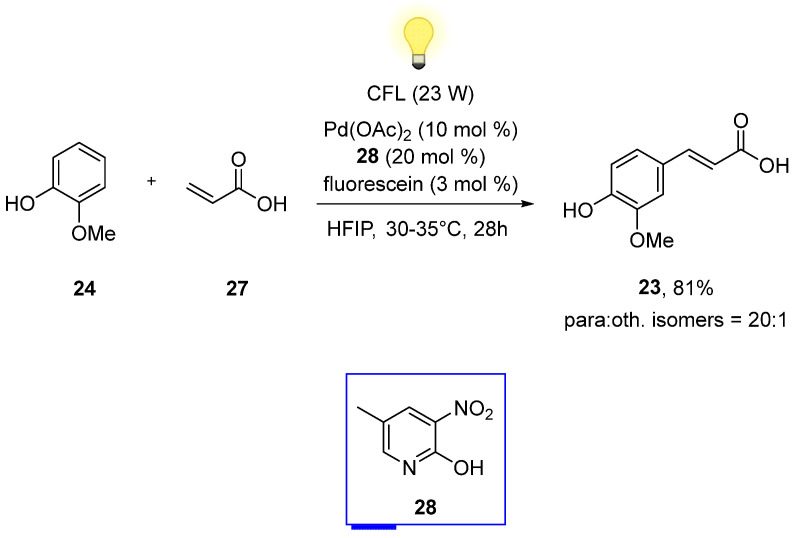
Preparation of ferulic acid **23** via photoredox Pd-catalyzed reaction. Ligand **28** is reported in the blue box.

Guaiacol (**24**), olefin **27**, Pd(OAc)_2_ (10 mol %), a pyridine-based ligand (**28**, 20 mol %), and fluorescein (3 mol %) were mixed in hexafluoroisopropanol (HFIP) as a solvent in the presence of a compact fluorescent lamp (CFL, 23W) at 30–35 °C for 28 h to afford **23** (Scheme 9). Good regioselectivity was obtained (20:1 for the *para*-directed olefination with respect to the -OH group), and the yield was good (81%). It turned out that fluorescein rendered regioselectivity alongside a high yield of the olefinated product. The inclusion of the pyridine-based ligand helped in accelerating the transformation. The role of light was both as an oxidant and activator [46]. In the same conditions, *p*-coumaric acid was obtained with a slightly lower yield (74%). Interestingly, the olefination of free phenol under thermal conditions (at 100 °C) using AgOAc as an oxidant and ligand **28** did not render any desired product. Therefore, the introduction of a photoinduced system to obtain regioselective olefination is very relevant.

**Scheme 10:**
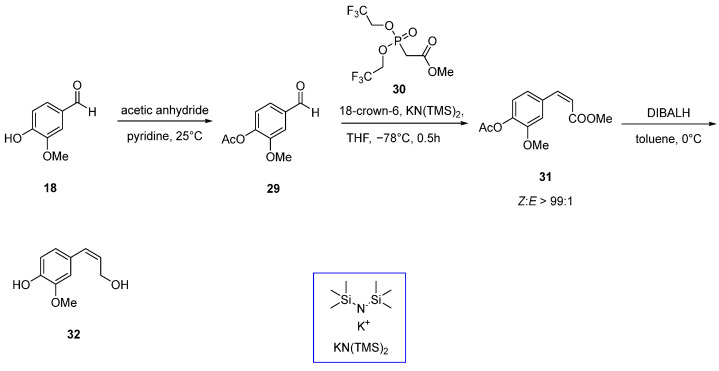
Preparation of (*Z*)-coniferyl alcohol **32** via using Still and Gennari’s modification of the Horner–Emmons olefination. The structure of potassium bis(trimethylsilyl)amide KN(TMS)_2_ is provided in the blue box.

A synthetic preparation for (*Z*)-coniferyl alcohol (**32**) has been reported as well (Scheme 10) [67]. The synthesis starts from vanillin (**18**), which is firstly protected at the phenolic group by its reaction with acetic anhydride to yield the acetyl-protected intermediate **29**. Subsequently, a Still and Gennari’s modification of the Horner–Emmons olefination using methyl bis(trifluoroethyl) phosphonoacetate **30** and KN(TMS)_2_/18-crown-6 as a base yielded (*Z*)-olefine **31**. The reduction mediated by DIBALH in toluene and the simultaneous removal of the acetyl group in toluene gave (*Z*)-coniferyl alcohol **32**. As stated by the authors, it is important that the crucial step (Still and Gennari’s olefination) is carried out at −78 °C in order to obtain almost exclusively (99%) the (Z) isomer. The (*E*)-isomer was formed even at −60 °C [67]. The authors reported a procedure for the synthetic preparation of lignin starting from **32**. NMR analysis of a synthetic lignin from (*Z*)-coniferyl alcohol indicated that the unsaturated sidechains in the resulting lignin retained their (*Z*)-geometry.

### 2.3. Sinapyl Alcohol

Sinapyl alcohol (**3**, Figure 1) is closely related to coniferyl alcohol, but with an additional methoxy group in its scaffold. Sinapyl alcohol possesses anti-inflammatory and antinociceptive (the process of preventing sensory neurons from detecting an unpleasant or harmful stimulus) properties [68]. Derivatives of sinapyl alcohol have demonstrated significant cytotoxic activities against BEL-7404 human hepatoma cells [7] and A-549, HL-60, and KB cancer cell lines [6]. Sinapyl alcohol exhibits antioxidant, antifungal, and antimicrobial activity [69].

**Scheme 11:**
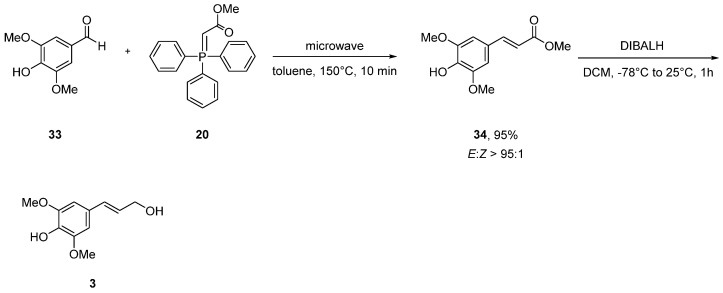
Preparation of sinapyl alcohol **3** via microwave-assisted Wittig reaction.

As for the preparation of coniferyl alcohol (cf. Scheme 6), sinapyl alcohol can be obtained via a microwave-assisted Wittig reaction starting from syringaldehyde **33** and Wittig ylide **20**, with an excellent yield (95%, regioselectivity *E*/*Z* > 95/1). The further reduction of **34** mediated by DIBALH in dichloromethane −78 °C provided sinapyl alcohol **3** (Scheme 11) [58].

**Scheme 12:**
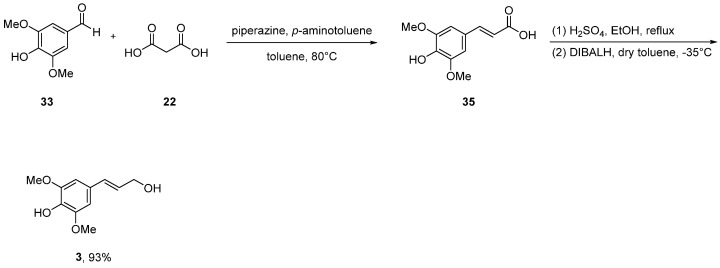
Preparation of sinapyl alcohol **3** via Knoevenagel condensation followed by Fischer esterification and reduction.

Alternatively, Knoevenagel condensation (with piperazine and *p*-aminotoluene as bases) between **33** and malonic acid **22** can provide **3** with additional steps (conversion of carboxylic acid **35** to ester via classic Fischer esterification and the final reduction by DIBALH in dry toluene at cold temperatures, Scheme 12) [70].

**Scheme 13:**
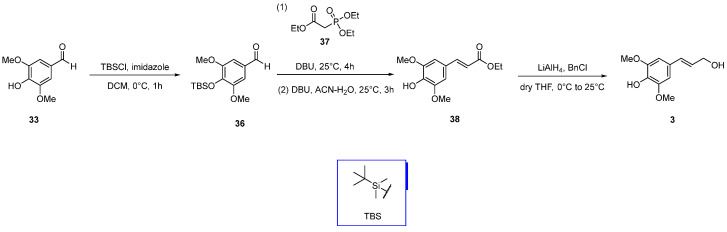
Preparation of sinapyl alcohol **3** via Horner–Wadsworth–Emmons (HWE) reaction. The tert-butyldimethylsilyl (TBS) protecting group is shown in the blue box.

An additional preparation (Scheme 13) [71] of **3** has been reported, employing triethyl phosphonoacetate (**37**) in a Horner–Wadsworth–Emmons (HWE) reaction. Firstly, syringaldehyde **33** is protected at the phenol functionality by using tert-butyldimethylsilyl chloride (TBSCl) and imidazole as a base in DCM at 0 °C to provide protected phenol **36**. Secondly, the Horner–Wadsworth–Emmons reaction takes place under 1,8-Diazabicyclo[5.4.0]undec-7-ene (DBU) conditions followed by deprotection of TBSCl (mediated by a mixture of acetonitrile/water), providing (*E*)-alkene **38**. This is lastly reduced to alcohol using a mixture of lithium aluminum hydride (LiAlH_4_) and benzyl chloride (BnCl) to give **3**.

**Figure 2:**
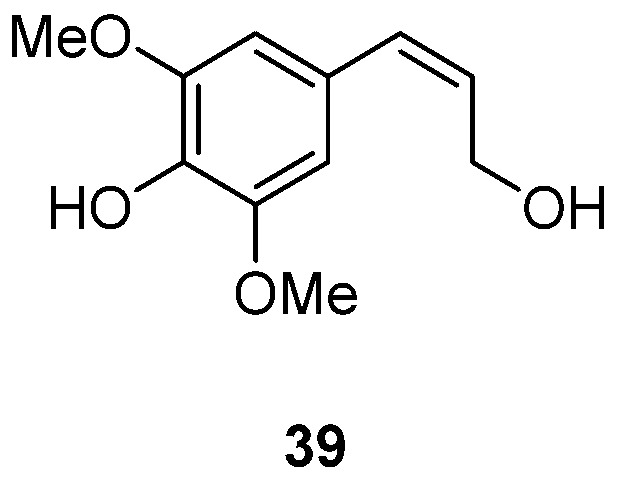
Chemical structure of (*Z*)-sinapyl alcohol (**39**). The synthetic pathway is identical to that described for (*Z*)-coniferyl alcohol **32**.

Still and Gennari’s modification of the Horner–Emmons olefination is useful to provide (*Z*)-sinapyl alcohol **39** (Figure 2) using the same conditions used for the preparation of (*Z*)-coniferyl alcohol **32** (cf. Scheme 10).

**Scheme 14:**
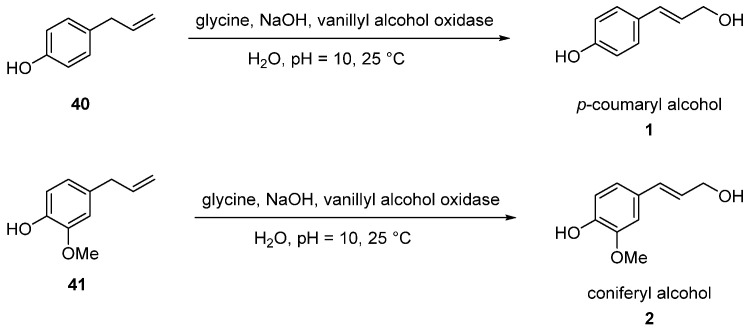
Greener process to prepare coumaryl alcohol and coniferyl alcohol from chavicol and eugenol [72].

A greener process to prepare coumaryl alcohol and coniferyl alcohol from chavicol **40** and eugenol **41**, respectively, was described by Fraaije et al. [72]. The conditions employed the following reagents: glycine, sodium hydroxide, and the enzyme vanillyl alcohol oxidase (Scheme 14). The solvent was water. Unfortunately, the authors did not report the chemical yields. Vanillyl-alcohol oxidase catalyzes the oxidation of 4-hydroxybenzyl alcohols, the oxidative deamination of 4-hydroxybenzylamines, and the oxidative demethylation of 4-(methoxymethyl)phenols.

Dell et al. [47] reported that through a borohydride reduction of the resulting mixed carbonic anhydrides, monolignols are effectively produced from the equivalent inexpensive and easily accessible cinnamic acids. Aqueous workup can remove the byproducts and produce high yields of clean products when the reaction conditions are carefully chosen.

**Scheme 15:**
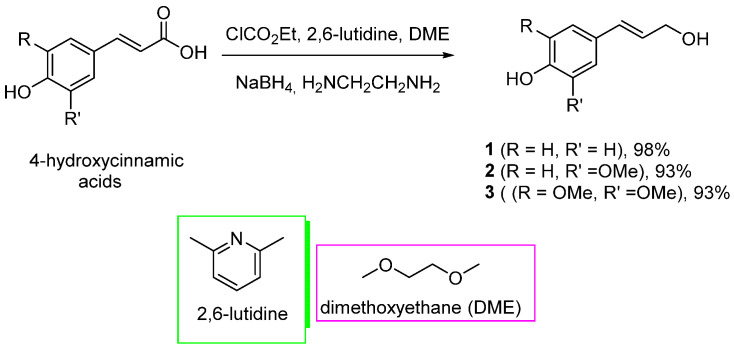
Facile and economical synthesis of the three canonical monolignols [47]. 2,6-lutidine is shown in the green box. DME is shown in the purple box.

In Scheme 15, the conditions (ethyl chloroformate, 2,6 lutidine as the base, and dimethoxyethane as the solvent) for the preparation of the three canonical monolignols are shown. The yields were outstanding for all three derivatives (above 93%).

The synthesis starts from inexpensive and readily available cinnamic acids using borohydride reduction of the corresponding mixed anhydrides. Ethylenediamine was chosen for deprotecting the phenol group, as we anticipated that the excess reagent and the corresponding byproduct could be removed via aqueous washing [47].

## 3. Unconventional Monolignols and Monolignol Analogues

Monolignol analogues refer to monolignol-like compounds that are not typically found in the natural lignin biosynthesis pathway, but they present a chemical structure similar to monolignols. These compounds can be synthesized or derived from alternative sources, offering unique chemical structures and properties that expand the possibilities for applications in various industries. Here, we explore a few examples of unconventional monolignols and their potential applications.

### 3.1. Caffeyl Alcohol, Monolignol

**Scheme 16:**
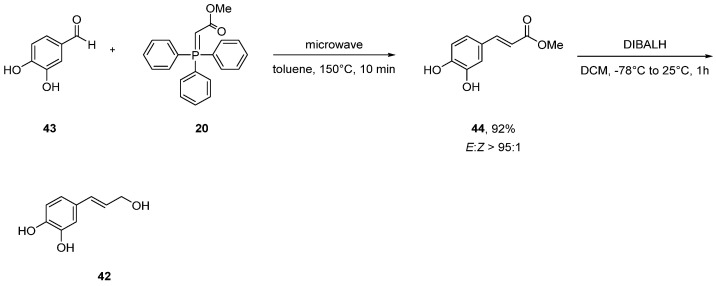
Preparation of caffeyl alcohol **42** via microwave-assisted Wittig conditions.

There are exceptions to the structure of lignin found in nature formed by the three canonical monolignols. For example, lignin in vanilla seed coats is formed almost exclusively by caffeyl alcohol (**42**, Scheme 16) units [26,27]. Caffeyl alcohol, also known as caffeic alcohol, is a phenolic compound, and it is an intermediate in the biosynthesis of coniferyl alcohol [73]. It is found in many plant species, including the seed coats of both monocot and dicot plants [1]. Caffeyl alcohol exhibits antioxidant properties and has been shown to have potential health benefits, including reducing inflammation and protecting against cardiovascular disease [74]. Moreover, it has been suggested that caffeic acid (the oxidized form of caffeyl alcohol) and its derivatives may have neuroprotective effects [75]. Oats, wheat, and rice are major sources of caffeic acid [76].

Caffeyl differs from coniferyl alcohol, as it has two free phenolic functions instead of only one. Carbon fibers based on caffeyl alcohol-based lignin have been prepared and described as linear and homopolymeric, in contrast to all known lignins, which are comprised of polyaromatic networks [1]. The chemical synthesis of caffeyl alcohol can be carried out via a microwave-assisted Wittig reaction, as seen for the coniferyl and sinapyl alcohols. Mixing 3,4-dihydroxybenzaldehyde **43** and ylide **20**, (*E*)-alkene **44** was obtained (92% yield and high regioselectivity), which was reduced by DIBALH in toluene to generate caffeyl alcohol **42** (Scheme 14) [58].

**Scheme 17:**
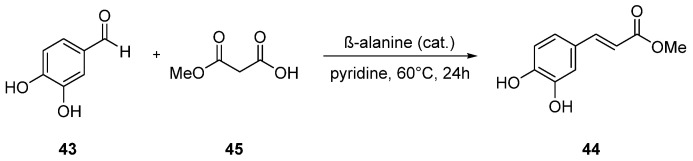
Preparation of (*E*)-alkene **44** via Doebner modification of the Knoevenagel reaction.

Another way to prepare (*E*)-alkene **44** is the Knoevenagel reaction (Doebner modification) between aldehyde **43** and monomethyl malonate **45**, employing β-alanine as a catalyst and pyridine as a solvent (Scheme 17) [77].

### 3.2. Iso-Sinapyl Alcohol, Non-Natural Monolignol Analogue

**Scheme 18:**
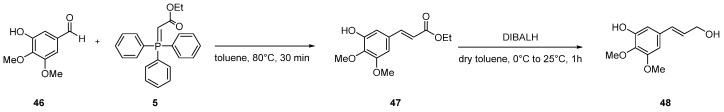
Preparation of iso-sinapyl alcohol (**48**).

Iso-sinapyl alcohol (**48**, Scheme 18) is the structural isomer of sinapyl alcohol (**3**). It is considered a monolignol-like metabolite, but it has not been previously identified in plants, nor has it been identified in the lignin biosynthetic pathway. Additionally, down-regulation of caffeic acid 3-O-methyltransferase (COMT) activity revealed the presence of a novel monolignol-like metabolite, identified as iso-sinapyl alcohol in switchgrass species. The structure of synthetically prepared iso-sinapyl alcohol was confirmed via ^1^H-NMR. Its synthesis took place from the Wittig reaction between 3-hydroxy-4,5-dimethoxybenzaldehyde (**46**) and ylide **5** to provide (*E*)-alkene **47**, which was then subjected to reduction by DIBALH in dry toluene to afford **48** [78].

### 3.3. 5-Hydroxyconiferyl Alcohol, Monolignol

**Scheme 19:**
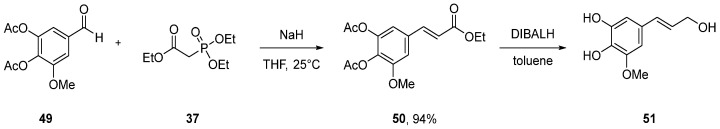
Preparation of 5-hydroxyconiferyl alcohol (**51**).

5-hydroxyconiferyl alcohol (**51**) differs from sinapyl alcohol based on its demethylated methoxy group in position 5. Its preparation starts from 5-hydroxyvanillin diacetate (**49**), which is converted (yield 94%) into the corresponding (*E*)-alkene **50** via Horner–Emmons olefination mediated by triethyl phosphonoacetate (**37**) in the presence of sodium hydride as a base. The reduction of ester to alcohol by DIBALH and simultaneous removal of acetyl groups yielded **51** (Scheme 19) [79].

As seen in the case of caffeyl alcohol, 5-hydroxyconiferyl alcohol (**49**) can be incorporated in lignin [79]. 5-hydroxyconiferyl alcohol is found in a variety of plant species, including maize [79] and sorghum [80]. It is produced through the action of enzymes known as hydroxycinnamoyl-CoA/shikimate hydroxycinnamoyl transferases (HCTs) on the precursor molecule, coniferyl alcohol. Lignins composed of caffeyl and 5-hydroxyconiferyl alcohol are linear in geometry and display characteristics that make them favorable for use in value-added products, such as lignin-based carbon fibers [81].

## 4. Concluding Remarks and Future Perspectives

**Figure 3:**
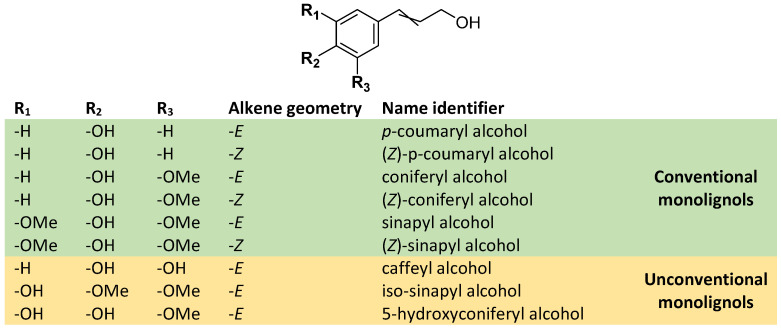
Overview of the monolignols described in this review. Conventional monolignols are displayed with a green background, while unconventional ones are shown with a yellow background.

In this work, the chemical synthesis of the three main monolignols (*p*-coumaryl alcohol, coniferyl alcohol, and sinapyl alcohol), two less-common monolignols (caffeyl alcohol and 5-hydroxyconiferyl alcohol), and one monolignol analogue (iso-sinapyl alcohol) was presented (cf. Figure 3). For each compound, different approaches for the chemical synthesis were provided, from the classical reactions (i.e., Wittig and Knoevenagel reactions) to more modern approaches (palladium-catalyzed olefination and photoinduced reactions), as summarized in Table 2.

If compared to other processes (depolymerization of lignin or monolignol biosynthesis), the bench synthesis of monolignols could bear the warning of not being a “green” process. On the other hand, the possibility of the discovery of an unexplored chemical space well outweighs these issues. For instance, in recent years, there has been considerable interest in lignin bioengineering to optimize the balance between reducing its recalcitrance for industrial processing and maintaining or enhancing the functional roles of lignin in plants, which could then be achieved through gene manipulation [82]. To align with these advances, methodologies for lignin characterization need to evolve as well, especially with regards to monolignols and monolignol conjugates [83]. Simultaneously, it is also important to further advance the mathematical models and computational simulations that have recently shown significant potential to further our understanding of the fundamental metabolism of lignin and related phenolic compounds [44]. Moreover, there are monolignol-like compounds that are not present in nature, like iso-sinapyl alcohol, that can be produced only through synthetic pathways. At present, the synthesis at bench scale of such derivatives has not gained much attention, but the possibility of obtaining interesting, versatile, and novel monomers must be considered. The yields for the described synthetic processes ranged from good to moderate. Additionally, the employment of green processes, as seen for the light-induced preparation of (*Z*)-*p*-coumaryl alcohol and the use of environmentally friendly solvents, could boost the employability of organic chemistry in this field.

Overall, the use of renewable raw materials for monolignol synthesis can help improve the sustainability of the process and reduce the environmental impact of the production of these important compounds. For instance, 4-hydroxybenzaldehyde, used in the synthesis of *p*-coumaryl alcohol, is one of the three isomers of hydroxybenzaldehyde. It can be found in the orchids *Gastrodia elata* [84]. Vanillin, an aldehyde-containing compound, is produced in tens of thousands of tons annually. Today, almost 15% of the world’s production of vanillin comes from lignin [85]. It is employed in the synthesis of coniferyl alcohol. Syringaldehyde is a phenolic aldehyde that is closely related to the vanillin found in fruits, nuts, and plants that synthesize lignin-related compounds [86], and it is the starting material for the preparation of sinapyl alcohol. Both starting materials and other reactants can be considered green. For example, malonic acid, employed in the synthesis of sinapyl alcohol **3**, is abundant in orange peels and juice [87]. Developing more energy-efficient synthetic methods can reduce these impacts. Microwave-assisted synthesis is an illustration of a green synthesis process for monolignols. In this process, the reaction mixture is heated, and the reaction is sped up by microwave irradiation. As a result, less time is needed for the synthesis process, and less energy is needed to heat the reaction mixture. Recently, new photoinduced transition-metal and external photosensitizer-free organic reactions have appeared in literature, and these methods are also applicable to the lignin model [88]. In particular the construction of C–C, C–O, C–N, C–I, C–B, C–F, and C–H(D) bonds is very valuable from an economic point of view. As for the cost of producing monolignol in large quantities, more research is needed to improve the efficiency and reduce the cost of production, given the fact that the research presented in this work is mostly academic and does not describe a large-scale synthetic pathway.

**Scheme 20:**
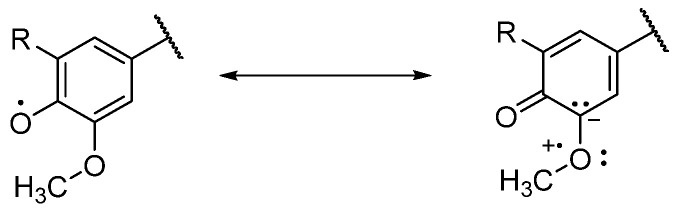
Resonance form of the phenoxy radical, a feature of the majority of the structures presented herein. The methyl group ortho to –OH is stabilizing the radical.

Moreover, there has been a renewed interest in lignin-based pharmaceutical manufacture in recent years as a result of the high demand for natural compounds. Monolignols and lignin monomers are polyphenols; as such, they present several groups, namely aliphatic hydroxyl groups, phenolic hydroxyl groups, methoxy groups, and carboxylate groups. These contribute to important biological activities; for instance, antioxidant activities [89]. In fact, *p*-coumaryl alcohol, coniferyl alcohol, and the monolignol derivative methyl (*E*)-3-(4-hydroxy-3,5-dimethoxyphenyl)acrylate have shown remarkable and higher antioxidant activities than butylated hydroxytoluene, which is typically used as a standard phenolic antioxidant [90]. Vanillin and ferulic acid, two lignin-derived components, have the potential to be employed as therapeutic agents against breast cancer [3], which is the most common cancer diagnosed in women. Ferulic acid was also reported to significantly lower plasma lipid and hepatic cholesterol levels and enhance antioxidant capacity in high cholesterol-fed rats [91]. More recently, lignin hydrogel patches have also demonstrated high ROS scavenging capabilities [92]. The antioxidant activity of monolignols, lignin monomers, and thus lignin itself results mostly from the presence of an ether oxygen positioned on the aromatic ring and in a ring system, which stabilizes the phenoxyl radical Ar-O∙ via stereoelectronic effects from the phenolic antioxidant (Ar-OH) (Scheme 20) [93]. Additionally, considerable studies have demonstrated that the o-dihydroxyl structure within the monolignols is essential for the free radical-scavenging and metal-chelating effects in hydroxy cinnamic acid derivatives. However, the role of the conjugated double bond at the 2,3-position on its antioxidant activity has not been verified [94]. It has also been shown that even though the antioxidant activity of these compounds is affected by the double bond, this structure feature alone is rarely responsible for the bioactivity of these phenylpropanoids [95].

**Figure 4:**
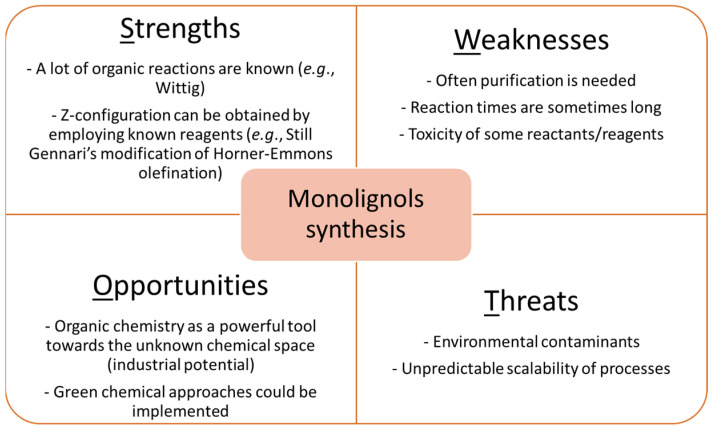
SWOT analysis of synthetic preparation of monolignols on the bench.

A SWOT (Strengths, Weaknesses, Opportunities, and Threats) plot is hereby presented (Figure 4) in order to highlight the pros and cons of using bench chemistry for the preparation of monolignols. Starting from the strengths, the chemistry used to prepare the described monolignols is well known, and there is a possibility of generating the desired configuration at the alkene site (cf. Still and Gennari’s modification for Z isomers).

The weaknesses are found in the purification stages and the toxicities of some reagents and solvents, which can have negative environmental and health impacts. For instance, the Wittig reaction uses solvents such as dichloromethane, which poses biological, chemical, and environmental hazards. Another major drawback of the Wittig reaction is that removing the phosphine oxide byproduct is sometimes difficult [96]. Similarly, the Knoevenagel reactions require harsh conditions, long reaction times, and the use of organic solvents, which cause environmental waste and pollution [97]. Moreover, it is clear based on this review that there are few studies dealing with the development of greener synthetic pathways for monolignols. Indeed, greener possibilities include (i) the biosynthesis of monolignols using microorganisms, though it is challenging to have all of the required metabolites, and this process has considerably higher costs; and (ii) the depolymerization of lignin; however, due to the complexity of this biopolymer, it is difficult to obtain monolignols, and when it is possible to obtain lignin monomers, these have low yields.

The opportunities mostly include the open chemical space, giving chemists room to explore novel and unconventional synthetic pathways and building blocks. The increasing focus on sustainability and the shift towards bio-based and renewable materials create opportunities for monolignol-derived products in various industries. This includes the demand for greener chemicals, biodegradable materials, and environmentally friendly alternatives. Threats are represented by the non-environmentally friendly nature of some chemicals, such as DIBAL-H, which are widely used in the above-described syntheses. Yet, we have shown that this last point could be mitigated using microwave-assisted or light-induced synthesis, together with the use of renewable starting materials. Overcoming the cost and performance advantages of these traditional methods can be a significant challenge.

In conclusion, chemical synthesis pathways offer the advantages of scalability and customization, allowing for the production of monolignols with specific chemical structures to suit various applications. Significant advancements have been made in optimizing reaction conditions, catalysts, and process efficiency to improve the yield and selectivity of monolignol synthesis.

At the same time, our review can inspire process chemists and medicinal chemists to develop modified building blocks and alternative strategies to synthesize monolignols. Moreover, this manuscript offers an overview for pharmaceutical chemists and biologists to prepare the substrates and reagents necessary for their studies in the laboratory, such as the synthesis of drugs and the study of biosynthesis pathways [2,98].

## Figures and Tables

**Table 1:** Overview of the chemical structures described in this review.

Structure	Identification Code #	IUPAC Name
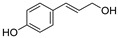	**1**	(*E*)-4-(3-hydroxyprop-1-en-1-yl)phenol
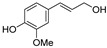	**2**	(*E*)-4-(3-hydroxyprop-1-en-1-yl)-2-methoxyphenol
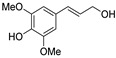	**3**	(*E*)-4-(3-hydroxyprop-1-en-1-yl)-2,6-dimethoxyphenol
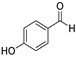	**4**	4-hydroxybenzaldehyde
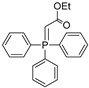	**5**	Ethyl 2-(triphenyl-λ5-phosphaneylidene)acetate
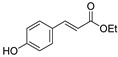	**6**	Ethyl (*E*)-3-(4-hydroxyphenyl)acrylate
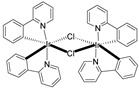	**7**	Chlorobis(2-phenylpyridine)iridium(III)dimer
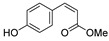	**8**	Methyl (*Z*)-3-(4-hydroxyphenyl)acrylate
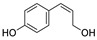	**9**	(*Z*)-4-(3-hydroxyprop-1-en-1-yl)phenol
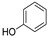	**10**	Phenol
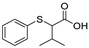	**11**	3-methyl-2-(phenylthio)butanoic acid
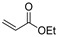	**12**	Ethyl acrylate
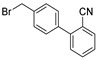	**13**	4′-(bromomethyl)-[1,1′-biphenyl]-2-carbonitrile
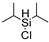	**14**	Chlorodiisopropylsilane
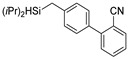	**15**	4′-((diisopropylsilyl)methyl)-[1,1′-biphenyl]-2-carbonitrile
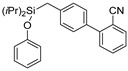	**16**	4′-((diisopropyl(phenoxy)silyl)methyl)-[1,1′-biphenyl]-2-carbonitrile
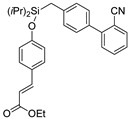	**17**	Ethyl (*E*)-3-(4-((((2′-cyano-[1,1′-biphenyl]-4-yl)methyl)diisopropylsilyl)oxy)phenyl)acrylate
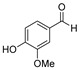	**18**	4-hydroxy-3-methoxybenzaldehyde
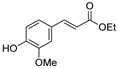	**19**	Ethyl (*E*)-3-(4-hydroxy-3-methoxyphenyl)acrylate
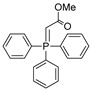	**20**	Methyl 2-(triphenyl-λ5-phosphaneylidene)acetate
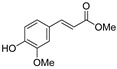	**21**	Methyl (*E*)-3-(4-hydroxy-3-methoxyphenyl)acrylate
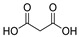	**22**	Malonic acid
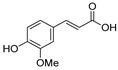	**23**	(*E*)-3-(4-hydroxy-3-methoxyphenyl)acrylic acid
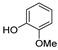	**24**	2-methoxyphenol
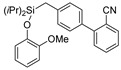	**25**	4′-((diisopropyl(2-methoxyphenoxy)silyl)methyl)-[1,1′-biphenyl]-2-carbonitrile
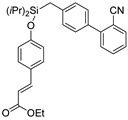	**26**	Ethyl (*E*)-3-(4-((((2′-cyano-[1,1′-biphenyl]-4-yl)methyl)diisopropylsilyl)oxy)phenyl)acrylate
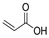	**27**	Acrylic acid
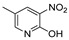	**28**	5-methyl-3-nitropyridin-2-ol
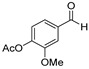	**29**	4-formyl-2-methoxyphenyl acetate
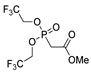	**30**	Methyl 2-(bis(2,2,2-trifluoroethoxy)phosphoryl)acetate
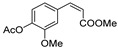	**31**	Methyl (*Z*)-3-(4-acetoxy-3-methoxyphenyl)acrylate
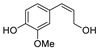	**32**	(*Z*)-4-(3-hydroxyprop-1-en-1-yl)-2-methoxyphenol
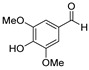	**33**	4-hydroxy-3,5-dimethoxybenzaldehyde
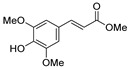	**34**	Methyl (*E*)-3-(4-hydroxy-3,5-dimethoxyphenyl)acrylate
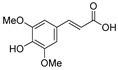	**35**	(*E*)-3-(4-hydroxy-3,5-dimethoxyphenyl)acrylic acid
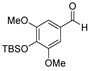	**36**	4-((tert-butyldimethylsilyl)oxy)-3,5-dimethoxybenzaldehyde
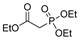	**37**	Ethyl 2-(diethoxyphosphoryl)acetate
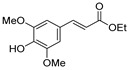	**38**	Ethyl (*E*)-3-(4-hydroxy-3,5-dimethoxyphenyl)acrylate
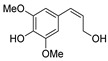	**39**	(*Z*)-4-(3-hydroxyprop-1-en-1-yl)-2,6-dimethoxyphenol
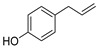	**40**	4-allylphenol
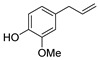	**41**	4-allyl-2-methoxyphenol
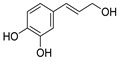	**42**	(*E*)-4-(3-hydroxyprop-1-en-1-yl)benzene-1,2-diol
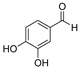	**43**	3,4-dihydroxybenzaldehyde
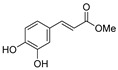	**44**	Methyl (*E*)-3-(3,4-dihydroxyphenyl)acrylate
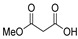	**45**	3-methoxy-3-oxopropanoic acid
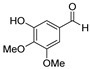	**46**	3-hydroxy-4,5-dimethoxybenzaldehyde
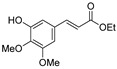	**47**	Ethyl (*E*)-3-(3-hydroxy-4,5-dimethoxyphenyl)acrylate
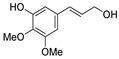	**48**	(*E*)-5-(3-hydroxyprop-1-en-1-yl)-2,3-dimethoxyphenol
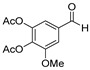	**49**	5-formyl-3-methoxy-1,2-phenylene diacetate
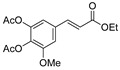	**50**	(*E*)-5-(3-ethoxy-3-oxoprop-1-en-1-yl)-3-methoxy-1,2-phenylene diacetate
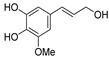	**51**	(*E*)-5-(3-hydroxyprop-1-en-1-yl)-3-methoxybenzene-1,2-diol
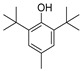	**52**	2,6-di-tert-butyl-4-methylphenol

**Table 2:** Summary of the processes used for the synthesis of monolignols, their chemical yield, and regioselectivity.

Synthesis of Monolignols				
Name of Monolignol	Reaction/Strategy	Yield	Regioselectivity	Reference
*p*-coumaryl alcohol (**1**)	Wittig reaction	90%	*E*	[51]
*p*-coumaryl alcohol (**9**)	Reacting **1** with with blue led light and an iridium catalyst **7**, Ir_2_(ppy)_4_Cl_2_	Not reported	*Z*	[53]
*p*-coumaryl alcohol (**1**)	Reagents: glycine, sodium hydroxide, and the enzyme vanillyl alcohol oxidase; substrate: chavicol	Not reported	*E*	[72]
coniferyl alcohol (**2**)	Wittig reaction	82%	*E*	[57]
coniferyl alcohol (**2**)	Microwave-assisted Wittig reaction.	Not reported	*E*	[58]
coniferyl alcohol (**2**)	Knoevenagel condensation to make ferulic acid, followed by reduction via DIBALH	Not reported	*E*	[59]
coniferyl alcohol (**2**)	Reagents: glycine, sodium hydroxide, and the enzyme vanillyl alcohol oxidase; substrate: eugenol	Not reported	*E*	[72]
(*Z*)-coniferyl alcohol **32**	Still and Gennari’s olefination	Not reported	*Z*	[67]
Sinapyl alcohol (**3**)	Microwave-assisted Wittig reaction.	95% (ester) Alcohol: not reported	*E*	[58]
Sinapyl alcohol (**3**)	Knoevenagel condensation followed by esterification and reduction	93%	*E*	[70]
Sinapyl alcohol (**3**)	Horner–Wadsworth–Emmons reaction (HWE)	Not reported	*E*	[71]
(*Z*)-sinapyl alcohol (**39**)	Still and Gennari’s olefination	Not reported	*Z*	[67]
caffeyl alcohol **40**	Microwave-assisted Wittig reaction.	92% (ester) Alcohol: not reported	*E*	[58]
caffeyl alcohol **40**	Knoevenagel reaction (Doebner modification) to prepare ester followed by reduction	Not reported	*E*	[77]
iso-sinapyl alcohol (**46**)	Wittig reaction	Not reported	*E*	[78]
5-hydroxyconiferyl alcohol (**49**)	Horner–Wadsworth–Emmons reaction (HWE)	94% (ester) Alcohol: not reported	*E*	[79]

## Abbreviations

ACN	Acetonitrile
AcOH	Acetic acid
AgOAc	Silver acetate
BnCl	Benzyl chloride
CAME	*p*-Coumaryl alcohol-γ-O-methyl ether
CFL	Compact fluorescence lamp
DCE	1,2-dichloroethane
DBU	1,8-diazabicyclo[5.4.0]undec-7-ene
DCM	Dichloromethane
DIBALH	Diisobutylaluminium hydride
DMAP	4-dimethyalaminopyridine
DME	Dimethoxyethane
EtOH	Ethanol
HAT	Hydrogen atom transfer
HWE	Horner–Wadsworth–Emmons
HFIP	Hexafluoroisopropanol
H_2_SO_4_	Sulfuric acid
(*i*Pr)_2_SiHCl	Chlorodiisopropylsilane
KN(TMS)_2_	Potassium bis(trimehylsilyl)amide
LiAlH_4_	Lithium aluminum hydride
MeOH	Methanol
Mg	Magnesium (metallic)
NaBH_4_	Sodium borohydride
NaH	Sodium hydride
NBS	N-bromosuccinimide
Pd(OAc)_2_	Palladium (II) acetate
PhCO_3_tBu	Tert-butyl peroxybenzoate
SET	Single electron transfer
TBAF	Tetra-n-butylammonium fluoride
TEA	Triethylamine
THF	Tetrahydrofuran
TFE	2,2,2-trifluoroethanol
18-crown-6	1,4,7,10,13,16-hexaoxacyclooctadecane
